# Increasing temperatures counteract the evolutionary consequences of fishing in model of Northeast Arctic Cod (*Gadus morhua*)

**DOI:** 10.1038/s41598-025-15394-x

**Published:** 2025-08-17

**Authors:** Henrik H. Jessen, Anders Frugård Opdal, Katja Enberg

**Affiliations:** Department of Biological Sciences, University of Bergen, 5006 Bergen, Norway

**Keywords:** Eco-evolutionary dynamics, Fishing pressure, Climate, IBM modelling, Evolutionary theory, Evolution, Ecology, Ecological modelling

## Abstract

Fisheries and climate warming are two stressors known to induce evolutionary changes in fish life histories. While their independent effects have been well documented, their interactive effects are less charted, although likely important for sustainable fisheries management and conservation strategies. We investigated the evolutionary responses of the Northeast Arctic cod stock (*Gadus morhua*) to warming temperatures and fishing pressure using a mechanistic modeling approach. Our individual-based simulation model incorporates explicit energy and oxygen budgets, and a simplified genetics framework to capture the complex interactions among traits governing energy acquisition/allocation and maturation schedules. Our results provide a theoretical basis for positive consequences for this particular cod stock in a warming climate. Warmer temperatures increased the aerobic scope, which reduced natural mortality. We found that if food availability and temperature are not linked, a warming climate leads to larger population sizes. By selecting for maturation at larger sizes, adaptation to warming climate at least partially counteracts the evolutionary consequences of fishing, namely smaller body sizes and earlier maturation. Our findings emphasize the benefits of adaptive management approaches, considering fish as evolving organisms and integrating ocean warming into fisheries management strategies.

## Introduction

Globally, fisheries compose a major source of food and income, and the sustainable development agenda^1^ point to the need for sustainable fishing and aquaculture to feed the world’s growing human population. In recent decades, the need for considering evolution has come into focus as it has become evident that marine species’ differ in their adaptive capacity, highlighting a need for evolutionary studies^2,3^. One commercially important fish species is Atlantic cod (*Gadus morhua*), which accounted for annual catches between 1.0 and 1.3 million tonnes from 2017-2020^4^, with the Northeast Arctic (NEA) cod stock alone accounting for annual catches around 800,000 tonnes in the decade between 2010 and 2020^5^. Fish stocks, such as the NEA cod, are generally managed through limiting the amount of fish caught through catch quotas, which in turn are based on estimates of stock size and fisheries reference points, and eventual long-term management plans. The capacity for evolution is generally ignored in such management schemes, possibly leading to mismanagement^6^. This is unfortunate, as both fisheries and climate warming are known to induce evolutionary changes in fish populations^7–11^. The mechanism of fisheries-induced evolution is straightforward: an additional source of mortality, frequently selecting against specific phenotypes. In contrast, climate warming impacts a wide range of processes, from internal physiology to ecosystem interactions, making its effects less direct and impacting all individuals in a population.

Two much studied effects are the influence of either fisheries or climate warming on fish life histories, both typically selecting for life history strategies characterized by higher growth rates, earlier maturation, and shorter lifespans^3,12–15^. However, little is known about the concurrent impacts of the two, despite the likelihood of both stressors intensifying in the coming years^16,17^. Do they amplify each others impacts, leading to intensification of their effects? Alternatively, does evolutionary adaptation in response to one stressor mitigate the impacts of the other stressor as well? Furthermore, most studies on evolution in response to climate warming have focused on temperate or warmer regions, while responses in species inhabiting the coldest edge of their thermal niche may show different patterns, especially once fisheries are also accounted for^18,19^.

Fisheries, even a non-selective one, will cause life-history evolution due to the increased mortality^3,20–23^. However, most fisheries are selective, disproportionately targeting specific life-history traits, such as size, maturity status, behavior, or location. Over time, fish populations are expected to evolve life-histories that minimize the associated increase in mortality risk. This implies earlier maturation and smaller size-at-age, as the targeted removal of larger individuals lessens the benefit of late maturation^7,10,20,24,25^. As such, maximising lifetime fecundity when fisheries are introduced, poses a trade-off between a life-history evolved towards natural mortality, which is typically highest for small individuals, and that dictated by fisheries mortality, which is typically highest for larger individuals^26^.

The selectivity of fisheries, however, depends on the gear type in use. In the case of NEA cod, the majority of fish are caught by trawling^5,13,27^ - a method that is designed to catch any encountered fish above a certain size, determined by the mesh size^26,28^. This enhances the type of selection mentioned above by further reducing the benefit of late/large maturation. Another important gear is the gillnet, which has a bell-shaped selectivity curve - selecting for a targeted size range while catching less of those smaller or larger than this range^26,29,30^. Gillnets therefore provide a size refugium: if the fish grow sufficiently large, their chances of being caught in the fisheries are significantly reduced, potentially favoring the strategy of rapidly growing through the risky size-range, delaying maturation in order to make use of the disproportionately larger fecundity of larger fish^31,32^. In comparison, the use of gillnets may theoretically favor later maturation compared to an unfished population, whereas trawling fisheries are expected to favor earlier maturation^13^.

Climate change is a term used to describe the collective consequences of human expansion and industrialization. For Atlantic cod, the consequence that has received the most attention is undoubtedly increasing ocean temperatures, which are shown to affect growth^33,34^, life-history^35^ and behavior^36^ of cod. This is somewhat expected, considering that temperature affects practically all biological processes and reactions, including metabolic rates, especially in ectotherms like fish^15,37^.

Atlantic cod is found across the North Atlantic, at a wide range of temperatures, from -1 to $20^{\circ }$C, although it is most commonly found between 0 to $12^{\circ }$C^38–40^. This, combined with its economic importance, has led to a large body of studies on e.g. consequences of fisheries^13,41,42^, food web dynamics^43,44^, migratory patterns^45–47^, long term population dynamics^27^ and responses to climate change^19,48^. This makes it an ideal species to model temperature effects. Moreover, temperature has previously been credited for nearly all of the differences in growth rates^49^, and populations from warmer regions do generally have faster maturation schedules^50^.

This trend towards smaller sizes and faster pace of life in warmer conditions, referred to as the Temperature Size Rule, is frequently attributed to reduction of aerobic scope^15,37^, which is assumed to decrease as temperature rises. Recently this assumption has been questioned^51,52^ and populations residing in the colder end of their thermal niches may in fact experience an increase in aerobic scope (as maximum metabolic rate increases faster than standard metabolic rate) and subsequent energy availability with increasing temperatures^53^, making generalisation difficult.

An evolutionary mechanistic modeling framework can offer an effective first-approach to unraveling complex interplay among various factors, commonly unattainable from the field. Instead of positing direct stressor effects on life histories, this framework allows life histories to emerge from the interactions of traits that optimize lifetime reproductive output. By adopting this approach, we not only avoid assuming static responses to stressors, but we also gain insights into the underlying mechanisms responsible for the observed changes in life histories.

This study builds upon the foundation laid by Jessen et al.^54^ in their model for NEA cod. Our objective is to further refine and expand this model to estimate the evolutionary responses of this cod population to concurrent warming temperatures and fisheries pressures.

By including both climate warming and fisheries pressure, we aim to fill a knowledge gap regarding how these changes might interact to shape life history consequences in the coldest tolerable regions for Atlantic cod, providing insights into the adaptive strategies we might expect in these populations.

## Model description

### Overview

**Figure 1:**
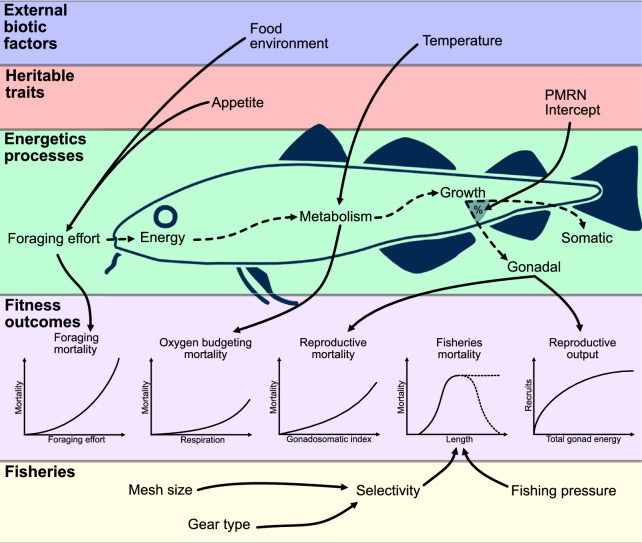
Conceptual overview of critical processes. Solid arrows indicate direct influence, and dashed arrows indicate the energy flow. The shaded ’%’ area represents the proportion of energy dedicated to somatic vs. gonadal growth. Figure is modified from Jessen et al.^54^, with mortality relations modified from Holt and Jørgensen^35^.

We utilise an individual-based, mechanistic model containing heritable traits and emergent evolution with explicit energy and oxygen budgets which impact growth, survival, and reproduction of the fish. The model has been parameterized for Northeast Arctic (NEA) cod, described in detail in Jessen et al.^54^. In this paper, we will present a simpler overview (Section Summary of processes), and highlight differences between Jessen et al.^54^ and this study. A visual overview of processes can be seen in Fig. 1.

### Purpose

The model aims to simulate evolutionary, phenotypic and demographic responses to climate warming, fisheries pressure and their interactions. To do so, we utilise a simplified genetics framework where we allow for inheritance of two genetic traits, which govern energy acquisition (appetite) and maturation schedule (Probabilistic Maturation Reaction Norm, PMRN, intercept), respectively. Both traits are described in the next section. Trait combinations that yield higher lifetime reproductive output will be passed on between generations more successfully than those that reduce lifetime reproductive output. Over time, this changes average life history to be centered around optimal trait combinations, mimicking evolution by natural selection.

### Summary of processes

For each scenario tested, the model started with the initial population, and was run 20 times for 2000 years to act as replicates. Parameterization was identical for replicates within each treatment, and differences result from stochastic processes (e.g. food availability; choice of parents; chance of maturation). The model operates in yearly steps, and is built around a bioenergetics framework, based upon the Wisconsin Bioenergetics Framework^55^, parameterized for NEA cod by Holt and Jørgensen^35^ and Jessen et al.^54^. Individual fish are simulated in annual time steps to live, reproduce and die as governed by their trait combinations, with a starting age of 1 year and a potential maximum age of 20 years.

Every individual has an intrinsic, inherited trait labelled Appetite which sets a desired annual energy intake ($J y^{-1}$). If possible, the individual will forage until it reaches this energy intake. The foraging effort needed to reach targeted energy intake is affected by density dependence, such that a larger population biomass will require the individual to increase its energy acquisition effort. Metabolic costs, which increase with increasing temperatures, are subtracted from the acquired energy, and the remainder is dedicated to growth. For immature individuals all remaining energy is allocated to somatic growth, but for mature individuals a set fraction of the energy is allocated to reproduction. This fraction increases as the fish ages post maturation^56^. In addition to the energy budget, the model also includes an oxygen budget, modified from Claireaux et al.^57^. This oxygen budget calculates oxygen consumption based on energy spent, and compare this to maximum oxygen uptake (which increase with increasing temperatures) to provide an estimate of respiratory capacity, as well as an upper bound for energy expenditure.

The maturation schedule is described using a Probabilistic Maturation Reaction Norm (PMRN)^58^. Here, the likelihood of maturing increases as an individual grows in size or age. The intercept of the PMRN is an inherited trait, and directly influences the age/size at which the fish mature. The PMRN intercept describes when the reaction norm crosses the y-axis, and can thus be thought of as the theoretical size at which 50% of 0-year old fish would mature. A higher intercept value will generally correspond to later maturation at a larger size. For more information on PMRN please refer to Dieckmann and Heino^58^.

The modelled recruitment, the addition of new individuals to the population, is based on the total reproductive investment of the population in any given year, here measured as total egg production calculated from total energy dedicated to gonads. The number of recruits (1-year-olds) is calculated using the Beverton-Holt recruitment function^59^, and once added these are treated as any other individual in the population. As such, maternal effects are not considered in this model. For every recruit, two parents are selected among the mature population for inheritance. Parents are selected randomly, but weighted by individual gonad weight in the year, so that individuals that have the largest gonad mass have higher probability of being selected as parents. For every recruit, the values of the two inherited traits are set based on the mid-parental values, with added stochasticity. We also check the resulting emergent heritability in the model to make sure it lies around 0.2, in line with typical values for life history traits^9,60,61^.

Mortality in the model is divided into 6 components, previously parameterized for NEA cod by Holt and Jørgensen^35^, as follows: 1) Fixed mortality, which is the size independent mortality experienced by all fish, accounting for e.g. sickness and disease. 2) Predation mortality, which is the likelihood of getting eaten. This decreases with size, and is used to scale other sources of natural mortality, since it is assumed that death from predation is the primary cause of natural death. 3) Foraging mortality, which is the mortality associated with food acquisition. This mortality increases with foraging effort, reflecting the reduction in sheltering/hiding behavior, as described in Jessen et al.^54^. 4) Reproductive mortality, associated with decreased swimming capacity due to changes in form factor (larger gonads make the fish bulkier and less agile) and courtship behavior, which is assumed to scale positively with the gonadosomatic index. 5) Respiration mortality, which is mortality from decreased respiratory capacity in relation to respiratory demand, essentially exhaustion, increasing as oxygen consumption approaches maximum oxygen uptake. 6) Fishing mortality, which is mortality caused by the removal of individuals by fishers. Fishing mortality was not included in Jessen et al.^54^ and is described in detail below.

The driver of evolution in the model is the optimization of lifetime reproductive output, centered on the trade-off between energy acquisition and mortality. Increases in lifetime reproduction results from trait combinations that allow for more energy to be invested in gonadal development without compromising or even increasing survival. These trait combinations will be favored by the simulated selection process in the model, and thus become more prominent, while traits that decrease lifetime reproductive output will become less prominent. Population demography emerge as a consequence of these changes over time, as individual trait combinations are expressed with phenotypic variation around heritable genetic values, resulting in individual life-histories.

Unlike in the original model by Jessen et al.^54^, the initial gonadal allocation upon maturing is not an evolving trait in the current model. This change was made primarily for two reasons. Firstly, the trait proved unimportant in the first application of the model Jessen et al.^54^, likely because it was not strongly selected for. Secondly, we found that during model parametrization, it was not possible to attain realistic life-histories for the most extreme climate warming scenarios with two evolving traits that both alter the onset of maturation and shape of growth curves. We tried to adjust the model to work within specific temperature ranges and noticed that when we changed certain parameters that usually influence maturation schedules, we could potentially alter the PMRN intercept and the age/length at which maturation occurs. However, if we allowed unlimited allocation, the model would adapt by directing all energy to reproduction once it matured, leading to unrealistically sudden halts in somatic growth. Since the problem stemmed from the two maturation-related evolving traits, we decided to keep evolving PMRN intercept as it is a more intuitive measure of maturation schedule and fix the trait that previously provided no explanatory value.

### Fisheries

In the real world, fishing mortality and gear type varies with season and location. When NEA cod is feeding in the Barents Sea, the fishery utilizes trawling nets, leading to a sigmoidal selection curve. In late winter/early spring mature individuals migrate south to spawning grounds along the Norwegian coast. Here, the fishery is dominated by gillnets, leading to a bell-shaped selection curve. Currently, around 30% of landed NEA cod are caught by gillnets, primarily at the spawning ground, while around 70% are caught by trawl, primarily in the Barents Sea^5^.

Because our model does not resolve seasons and geography, we need to simplify these spatiotemporal dynamics. In the model, we thus allow any individual to be caught by any gear type by randomly exposing 70% of individuals to the trawling fishery, and 30% to the gillnet fishery. Fisheries and gear selectivity is simulated by assigning each individual a selectivity coefficient between 0 and 1, based on size. At 1, the individual experiences the maximum fishing mortality $F_{max}$, while at 0 it experiences no fishing mortality. Fisheries mortality, *F* is then calculated as

1 $$\begin{aligned} F = F_{max} * U(L) \end{aligned}$$

where *U*(*L*) is the selectivity coefficient at size *L*.

The bell-shaped gillnet selectivity, $U_G (L)$, is modelled as a Gaussian function, as in Jørgensen et al.^13^, around the targeted size at maximum selectivity, $L_{max}$,

2 $$\begin{aligned} U_G (L) = exp(\frac{- (L(i) - L_{max})^2}{2 \sigma ^2}) \end{aligned}$$

where *L*(*i*) is the length of individual *i*, and $\sigma$ is the selectivity width parameter. Like Jørgensen et al.^13^, we used the findings of Huse and Soldal^26^ to determine $\sigma$. They found a mean width of 0.14, but we assume that mesh size regulations in actual fisheries would catch a wider range of sizes than one for a scientific study, and thus doubled this value: $\sigma = 0.28*L_{max}$, the same value used by Jørgensen et al. (2009). Internal testing did not find significant differences in results between these widths.

For the sigmoidal trawl selectivity, $U_T(L)$ also as seen in Jørgensen et al.^13^, we used the left half of the same Gaussian function, while for lengths above $L_{max}$ the selectivity coefficient was set to 1,

3 $$\begin{aligned} U_T(L) = {\left\{ \begin{array}{ll} U_G(L), & L(i) < L_{max}\\ 1, & L(i) \ge L_{max} \end{array}\right. } \end{aligned}$$

While traditionally logistic functions are used for trawling selectivity, we chose this method to make the different catch methods more directly comparable. Additionally, $L_{max}$ was set to 110*cm* based on the same ICES data^5^, where maximum fishing mortality was experienced around 11 years age.

### Climate warming

**Figure 2:**
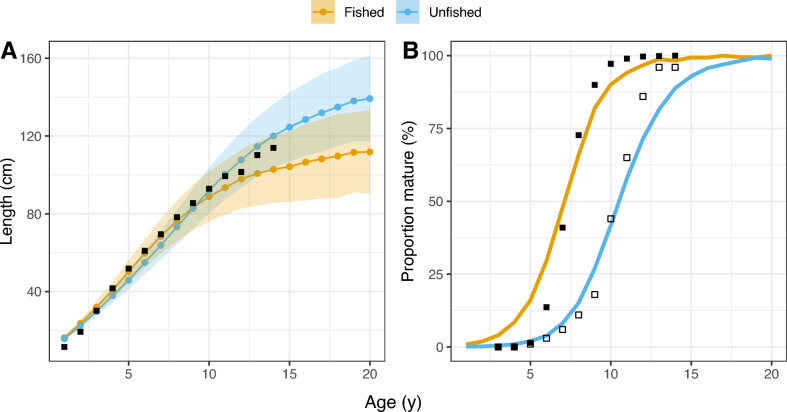
Stabilized populations compared to ICES data (squares)^5^. (**A**) Average length-at-age ±SD, compared to ICES averages from 2014 to 2021. (**B**) Average proportion-mature-at-age of the last 200 years of model runtime, compared to ICES averages from 2014 to 2021 (black squares) and 1946 (hollow squares).

Three warming scenarios are considered (Fig. 2), extrapolated from the emission scenarios presented in the 2021 report published by the Intergovernmental Panel on Climate Change^62^. These refer to IPCC scenarios SSP1, SSP2 and SSP3, which stabilise around mean Barents Sea surface temperatures of $4.8^{\circ }$C, $7.0^{\circ }$C and $12.4^{\circ }$ C respectively, within 100-500 years. In addition to these, a no-warming baseline scenario was also included. It is worth noting that the parametrisation of this model was done on a cold-water population of cod, and model assumptions become less reliable the farther away the scenarios move from present day temperatures. Particularly the warming scenario SSP3 predicting temperature increase of $8.4^{\circ }$C might be outside the reliable predictive range of our model, but this scenario is still included to test model performance in more extreme cases, and its reliability will be further expanded on in the discussion.

### Initializing the model

**Figure 3:**
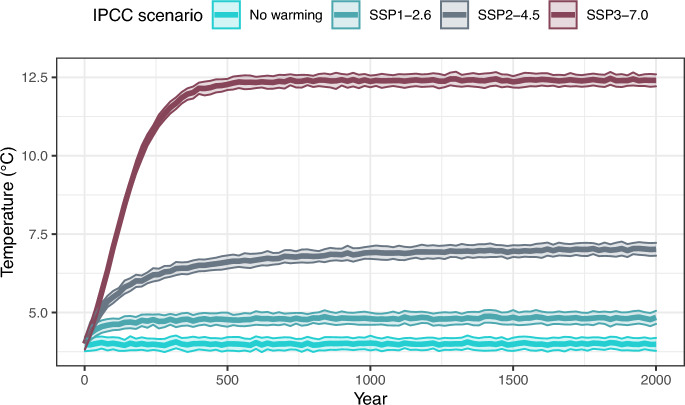
Temperature time-series used throughout the model runs ±SD based on data given by IPCC scenarios^62^.

To create the initial population, from which all model runs will start, we started by running the model once for 5000 years under a moderate level of fishing pressure, corresponding to a maximum fishing mortality, $F_{max}$, of 0.2 with temperature around $4^{\circ }$C. This allowed the population to reach an eco-evolutionary stochastic equilibrium, and produced a population size of 121.590 individuals that was used as a starting point for all our runs. To check the overall parameterization of the model, we also conducted an additional model run of 5000 years without fisheries, producing a second population of 330.617 individuals. The maturation patterns from these two populations were then compared to observed length-at-age and maturation patterns early (1946) and late (2014-2021) in the history of industrial fishing (Fig. 3). From here, all model runs were initiated with the population evolved to moderate fishing, which closely resembles the current (2014-2021) NEA cod population in terms of age and length at maturation.

## Results

**Figure 4:**
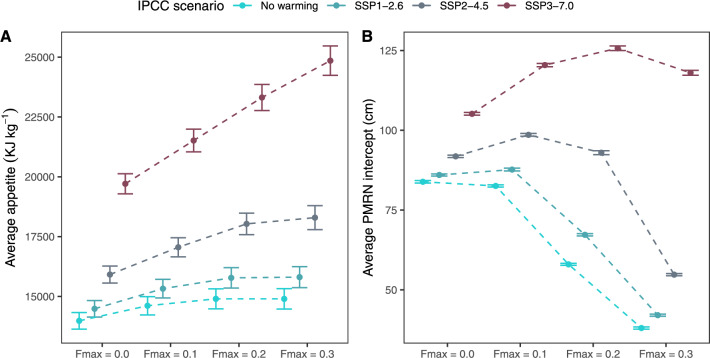
Resulting average genetic appetite and PMRN intercept ±SD at the end of the model’s runtime. Dashed lines visualise the effect of fishing pressure within warming scenarios.

We found that both increasing fishing pressure and increasing temperatures select for higher appetite (Fig. 4A, Appendix A, Fig A1). Evolution of PMRN intercept appeared more complex (Fig. 4B, Appendix A Fig A2): for the cooler temperature scenarios (no warming and SSP1), increase in fishing pressure selected for a reduction in intercept value, while for warmer SSP2 the value increased slightly from $F_{max} = 0.0$ to $F_{max} = 0.1$, before declining at higher levels of fishing pressure. For the warmest scenario (SSP3), the intercept value increased with fishing pressure until $F_{max} = 0.2$ and started to decline at the highest level of fishing pressure included in the model.

**Figure 5:**
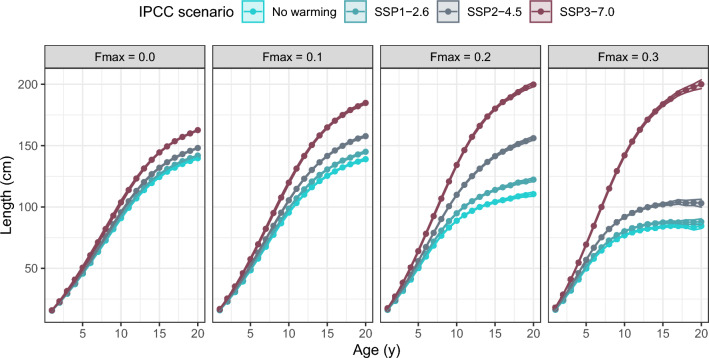
Resulting growth curves at the end of the models simulation time of 2000 years, showing average length-at-age ±SD.

Considering each fishing scenario separately (within each panel in Fig. 5), higher temperatures led to faster growth, and a higher relative length-at-age. However, when comparing each warming scenario in isolation (across panels in Fig. 5), we found that for the two coldest scenarios (no-warming and SSP1), increases in fishing pressure led to a decrease in asymptotic size, and generally smaller length-at-age across all ages above 5 years, with only a small increase below 5 years. In the warmest scenario (SSP3), asymptotic size increases with fishing pressure, while age distribution (Appendix B, Fig. B1) shifted towards younger individuals. This shift towards younger fish are also found for all other temperature scenarios (Appendix B, Fig. B1).

**Figure 6:**
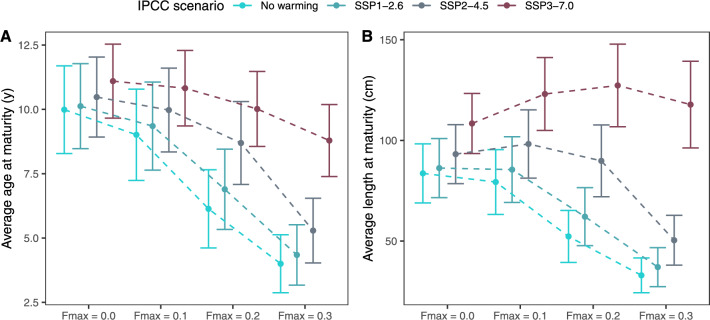
Average age/length at maturation ±SD at the end of the model’s runtime. Dashed lines visualise the effect of fishing pressure within warming scenarios.

Further, increased temperatures led to an increase in both age-at-maturity (Fig. 6A) and length-at-maturity (Fig. 6B). Increased fishing pressure led to a decrease in age-at-maturity for all temperature scenarios (Fig. 6A). Regarding the effect of temperature and fishing on the average length-at-maturity (Fig. 6B), this follows directly from the evolution of the PMRN-intercepts trait (Fig. 4B). Thus, when comparing Fig. 6A, B, we found that for the two warmest scenarios, an increase in fishing pressure led to a concurrent decrease in age-at-maturity (Fig. 6A) and increase in length at maturity (Fig. 6B). This follows from the increased growth rates associated with higher temperatures (Fig. 5), increasing the average length at age.

**Figure 7:**
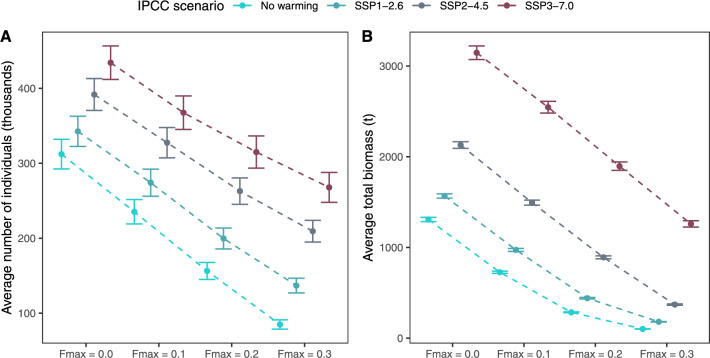
Average number of individuals and total population biomass at the end of the model’s runtime. Dashed lines visualise the effect of fishing pressure within warming scenarios.

For all combinations of warming and fishing, the population size measured both in total population biomass and total number of individuals, increased with warmer temperatures, and decreased with increased fishing pressure (Fig. 7, Appendix A Fig. A3 and A4). The warmest scenario (SSP3) in combination with the absence of fishing led to a 10-fold increase in population biomass (compared to no warming, $F_{max}=0.2$), while the total number of individuals increased by a factor of 3.5. On the other end of the spectrum, the no-warming scenario combined with the highest level of fishing pressure ($F_{max} = 0.3$) stabilised with a relative number of individuals of 70% and a relative population biomass of 34%, compared to the initial population, indicating that there are fewer individuals, and that each individual is smaller.

In all temperature scenarios except SSP3, increased fishing pressure led to an increase in predation mortality (Appendix C, Fig. C1), increased respiration mortality (Appendix C, Fig. C3) and increased reproductive mortality (Appendix C, Fig C4). Total mortality increased for all age groups with increasing fishing pressure, with the warmest scenario (SSP3) peaking at earlier ages, and then declining around age 8-10 (Appendix C, Fig C6.)

## Discussion

We used an individual-based model to investigate the eco-evolutionary consequences of concurrent climate warming and fisheries pressure for fish life histories and population size. Our results suggest that NEA cod adapting to increasing temperatures evolve towards faster growth, lower natural mortality and larger population biomass. Fishing mostly has the opposite effect, increasing natural mortality, selecting for smaller individuals and resulting in a smaller population size. This indicates that warming temperatures, at least for NEA cod, might have the potential to mitigate some of the effects of fisheries that are typically considered detrimental, at least in this simplified system with no climate effect on species interactions, spatial distribution, or food abundance.

The realism of our predictions with this simplified model is dependent on the accuracy of the physiological responses to temperature. Given that parametrisation and calibration was carried out with respect to NEA cod, an Arctic population, the farther temperature moves from current experienced range, the less reliable our parametrisation and resultant outputs become. Furthermore, the model assumes no changes in the ecosystem aside from fisheries and temperature, which particularly in the case of SSP3 seems improbable. Temperature rise of such magnitude is likely to considerably change the ecosystem, inducing physical changes (salinity, pH, $O_2$), changes in primary and secondary production, as well as in abundance and distribution of fish, marine mammals and birds^63^. Such changes are ignored in our analysis. Although Ecosystem models such as Atlantis^64–66^ or Ecopath with Ecosim^67^, might be better suited to anticipate general responses across whole ecosystems and could potentially serve as input for further evolutionary modelling in these much higher temperature ranges, their predictions will also grow more uncertain the farther away the conditions move from the base state^67^. In our model’s current form, we have low confidence in the accuracy of both assumptions and parametisation in the most extreme warming scenario (SSP3), and would advise against drawing conclusions for such high temperatures based on these results.

Our findings support the consensus that increased fishing mortality, particularly targeting larger individuals, selects for earlier maturation. As fisheries increase mortality, they decrease the likelihood of surviving to larger sizes/ages. By harvesting large individuals in particular, fisheries also drastically increase mortality for large fish, making it less beneficial to delay maturation to capitalize on the high fecundity of larger fish. These mechanisms have been extensively discussed in earlier research^3,10,13,24,32^, and so we won’t go into detail discussing them here. The results generated by this model rely on certain assumptions about the fisheries, namely the 70-30 split between trawl and gillnet, and the unadapting strategy employed by the fisheries. These assumptions are likely not strictly realistic, as fisheries strategies change based on continuous stock assessment^5^ and updated management strategies, but our conclusions do nevertheless show a theoretical basis for how fisheries management can interact with a warming climate to drive life-history evolution in NEA cod. Extending our model with adaptive management where fisheries strategies change continuously based on stock indices or trait distributions could be an interesting point of future research.

In response to warming, we see the opposite trend from fisheries, an evolution towards older age and larger size at maturation. This is somewhat opposite of other studies suggesting that the aerobic scope would decrease as temperatures increase^15^ and consequently lead to evolution towards a faster pace of life and smaller sizes^12^. However, in the case of NEA cod, which reside in the colder end of their thermal niche, increasing temperatures from the status quo initially leads to an increase in aerobic scope^35,53^, as lower temperatures suppress maximum metabolic rate. This leaves more energy available for growth, as well as to a decrease in natural mortality. This aligns with contemporary research predicting positive effects of warming on NEA cod^68^, where increasing temperatures come closer to optimal temperatures for Atlantic cod, and food availability is expected to increase as a result of retreating sea ice and increased primary production. Consequently, this study indicates that the combination of decreased mortality and increased energy availability, resulting from the increased aerobic scope, favors early growth and a delayed maturation schedule. While the model assumes certain temperature scenarios provided by the IPCC^62^, given the unidirectional response within all tested scenarios, our results do not appear to rely notably on the exact values of the warming trend.

A counterintuitive interaction between warming and fisheries, is the effect that fishing has on growth curves in warm scenarios, SSP2 and SSP3 (Fig. 5). Surprisingly, we observe an increase in length-at-age for older fish when fishing mortality is elevated in the warmest scenarios. Given that fisheries disproportionately target larger fish, this finding contradicts initial expectations. The mechanism behind this is the reduction in population biomass caused by the fisheries, and the subsequent reduction in population density. While fishing mortality exerts a direct mortality, it also provides a measure of relief from foraging mortality. While foraging mortality appears nearly constant between fishing scenarios (Appendix C, Fig. C2), the scenario with lower total population biomass allows for more energy acquisition. This holds true for all tested levels of fishing mortality in IPCC scenario SSP3, while in SSP2, the pressure from fisheries appears to overcome the benefits of larger sizes at $F_{max}=0.3$.

For population dynamics, it is unsurprising that elevated fishing mortality leads to a reduction in population size. However, our results indicate that the simultaneous reduction in mortality and increased energy availability accompanying warming may help mitigate this effect, at least for NEA cod. This aligns with earlier findings for North Sea cod by Neuheimer and Grønkjær^14^, who found that the increased growth from warming temperatures counteracted the effects of earlier maturation on length-at-age. While interesting, it should be noted that our model does not take into account trophic interactions or community dynamics. A growing body of research indicates that many species are changing their distribution to match their current thermal range, leading to a poleward shift^36,69,70^. These changes are already observed to be changing species composition and relative abundances of local species, while the consequences for ecosystem dynamics remain largely unknown^71,72^. Food availability in our model is unaffected by temperature – whether this is a realistic simplification or not is a complex question, and would be an interesting avenue for future research. As pointed out by Kortsch et al.^71^, the introduction of more generalist species might strenghthen connectivity of the trophic levels, aiding in energy transfer, but how this affects any one given species is beyond the scope of this study. Furthermore, it is unknown how retreating ice-cover around the North pole might affect spatial distribution of not only cod, but also key prey species such as capelin.

In summary, our findings provide a theoretical foundation for anticipating positive effects on the NEA cod stock in a warming ocean. While many factors, notably community interactions and trophic ecology, are not accounted for, NEA cod might experience a decrease in natural mortality and an increase in available energy due to increased aerobic scope and larger appetite. These changes could, at least partially, counteract the effects of fisheries, both in terms of population decline and evolutionary consequences, but does not appear to alter the effect fishing has on population age-structure. Our results thus show the importance of considering fish evolution, especially in response to warming and fisheries, in long-term management aiming to maximise sustainable yield.

## Supplementary Information

Supplementary Information 1.

Supplementary Information 2.

Supplementary Information 3.

Supplementary Information 4.

## Data Availability

All source code employed in this study, both for the IBM and data treatment, is readily available on Henrik H. Jessens GitHub repository: https://github.com/henrikhjessen/NEAC_ibm/tree/main
