# Supplementary material for: Increasing temperatures counteract the evolutionary consequences of fishing in model of Northeast Arctic Cod (Gadus morhua)
Source: Sci Rep. 2025 Aug 17;15:30039. doi: 10.1038/s41598-025-15394-x (PMC12358614; doi:10.1038/s41598-025-15394-x)
Supplement: Supplementary file 1 — Supplementary Information 1. [file 41598_2025_15394_MOESM1_ESM.pdf]

Appendix A: Changes in population average ±SD for (A1) appetite (A2) PMRN intercept (A3) total number of individuals and (A4) total population biomass.

Appendix B: Average proportion of the population (by count) from the last 100 years of model runtime, made up by individuals within each given age group, faceted for ease of comparison. (B1) Facets indicate warming scenarios, comparing fishing pressure. (B2) Facets indicate fishing pressure, comparing warming scenarios.

Appendix C: Contributions of sources of mortality based on age, averages of last 100 years of runtime was used, ±SD. (C1) Predation (C2) Foraging (C3) Respiration (C4) Reproduction (C5) Fisheries (C6) Total mortality
