# Supplementary figures and images for: Increasing temperatures counteract the evolutionary consequences of fishing in model of Northeast Arctic Cod (Gadus morhua)
Source: Sci Rep. 2025 Aug 17;15:30039. doi: 10.1038/s41598-025-15394-x (PMC12358614; doi:10.1038/s41598-025-15394-x)

IPCC scenario    No warming    SSP1–2.6    SSP2–4.5    SSP3–7.0

**A1**

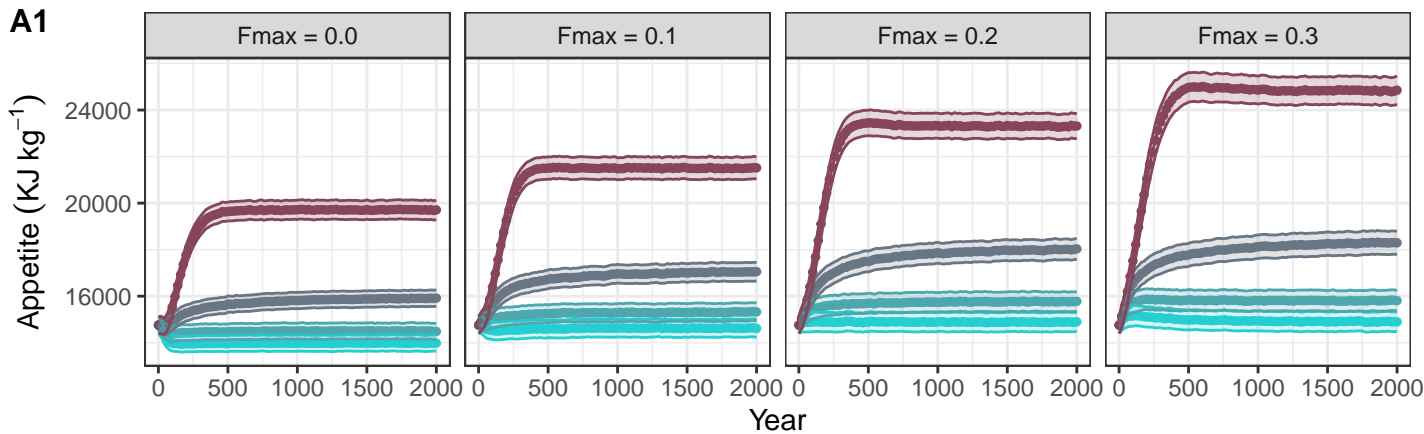

**A2**

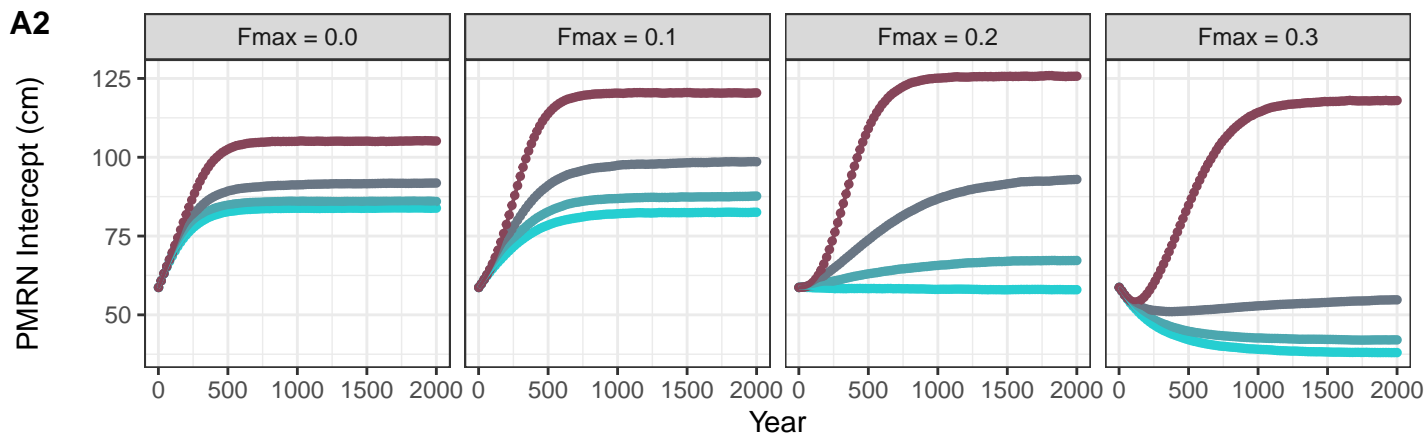

**A3**

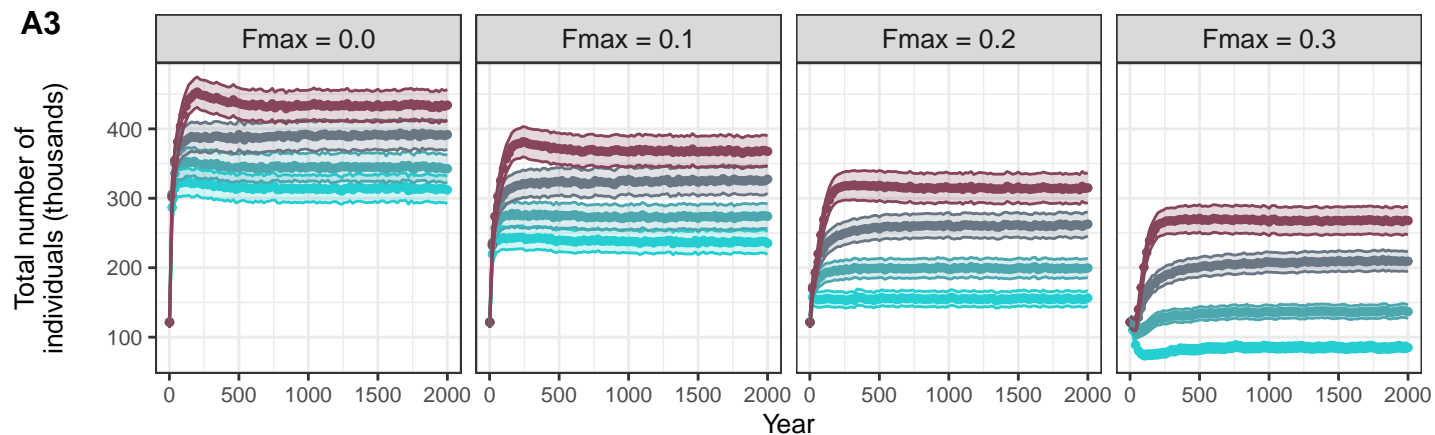

**A4**

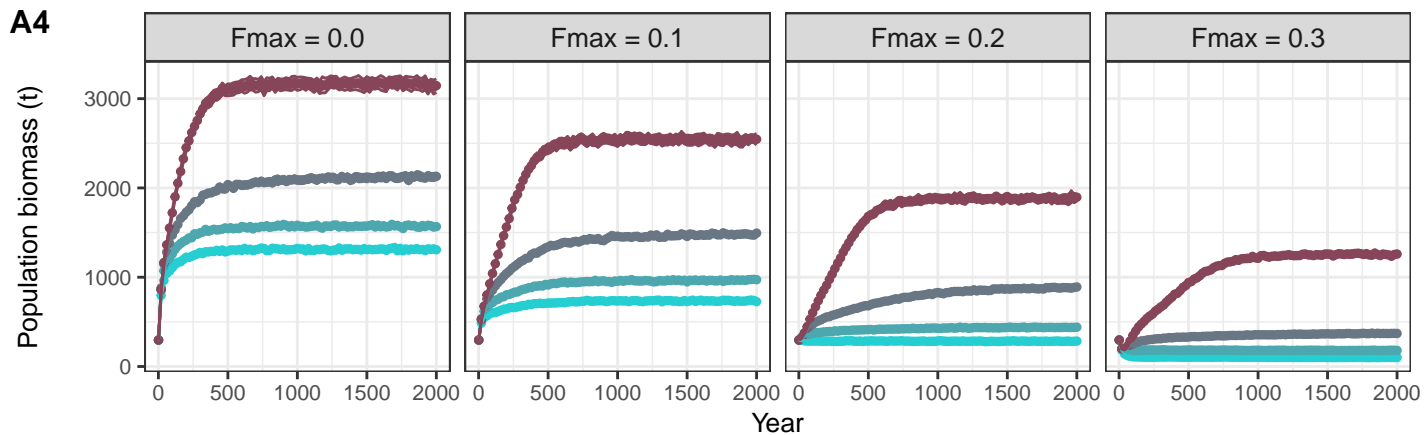

Supplement: Supplementary file 2 — Supplementary Information 2. [file 41598_2025_15394_MOESM2_ESM.pdf]

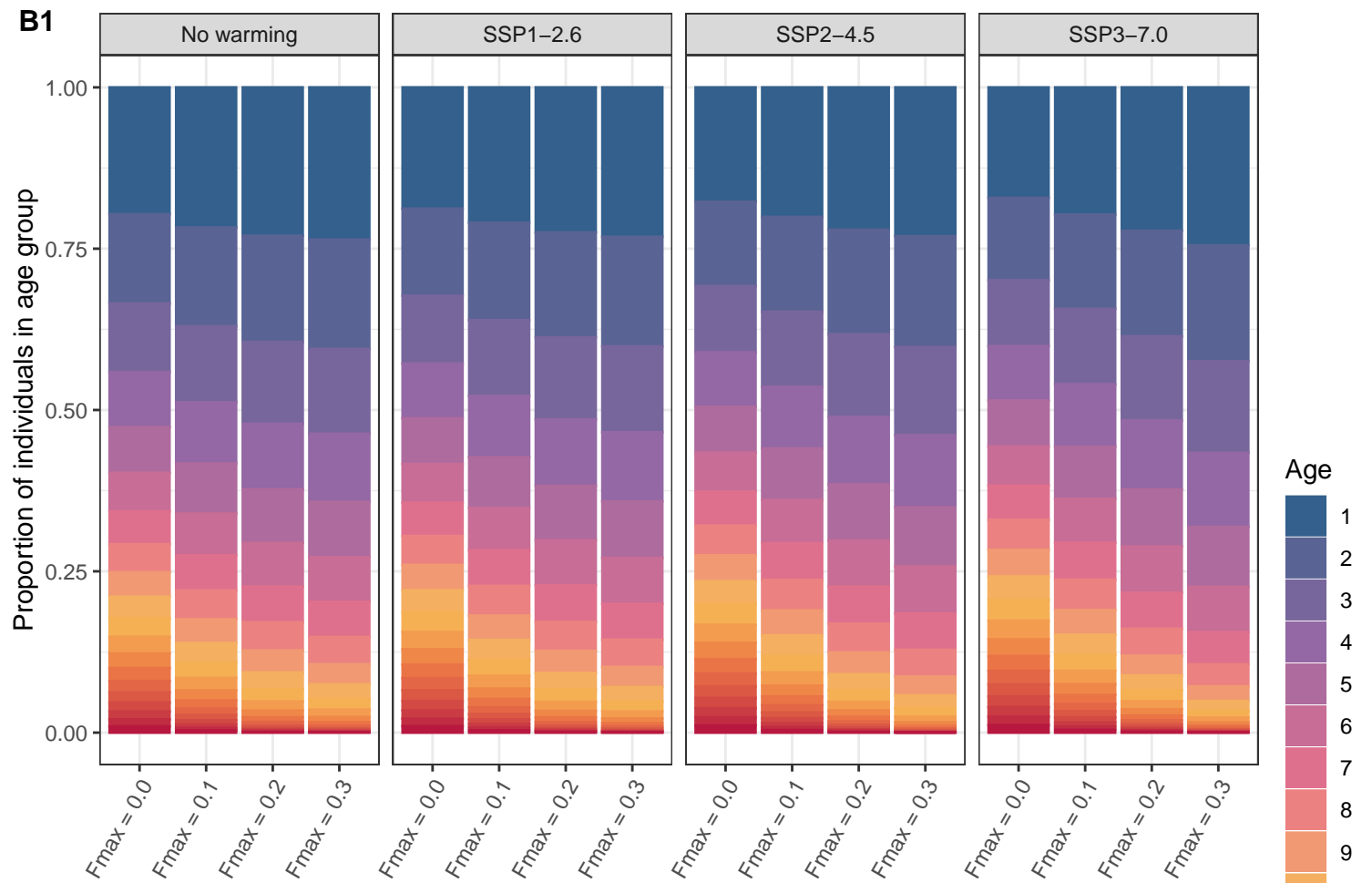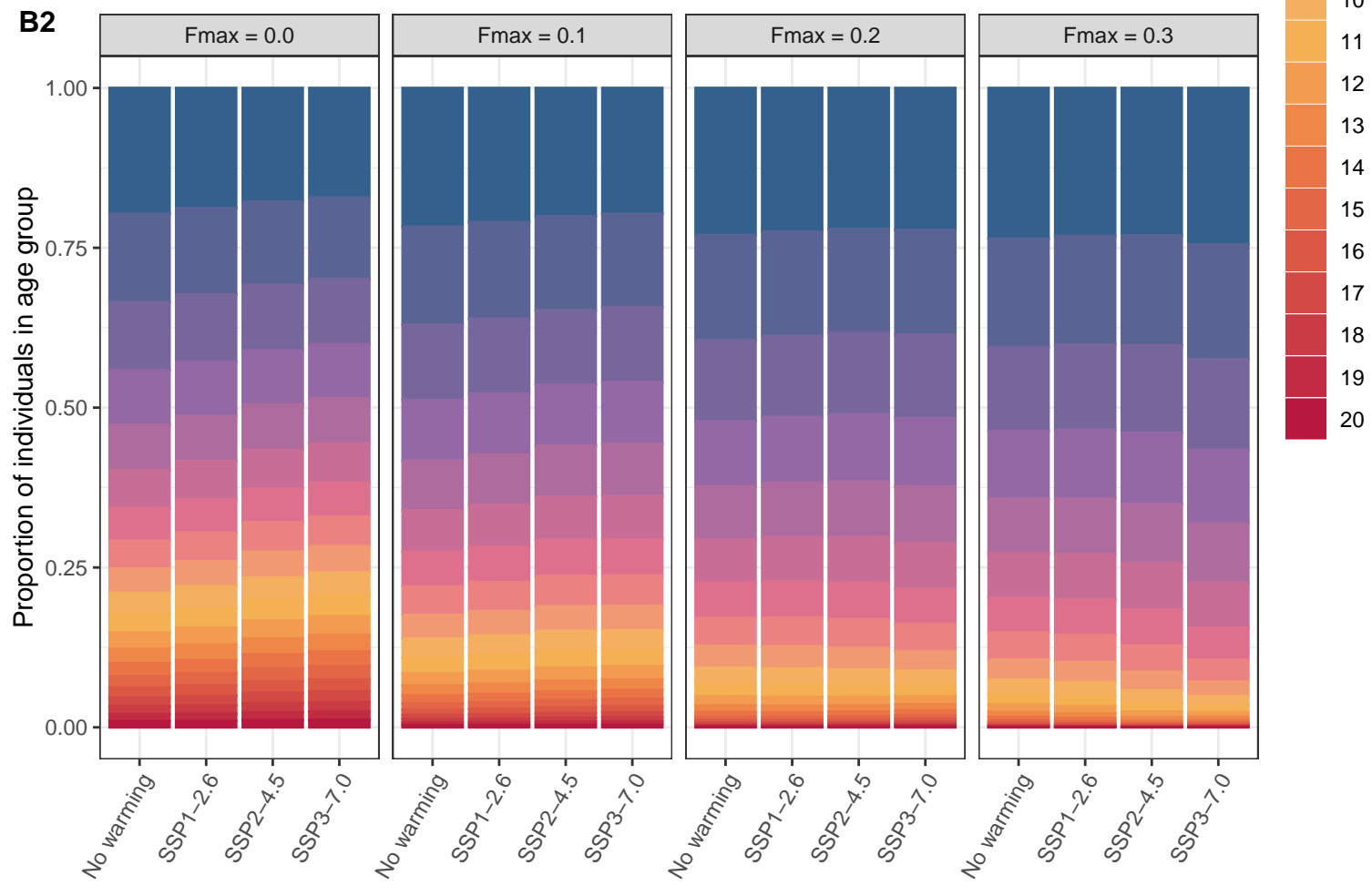

Supplement: Supplementary file 3 — Supplementary Information 3. [file 41598_2025_15394_MOESM3_ESM.pdf]

IPCC scenario

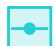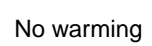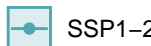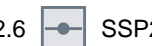

No warming    SSP1-2.6    SSP2-4.5    SSP3-7.0

**C1**

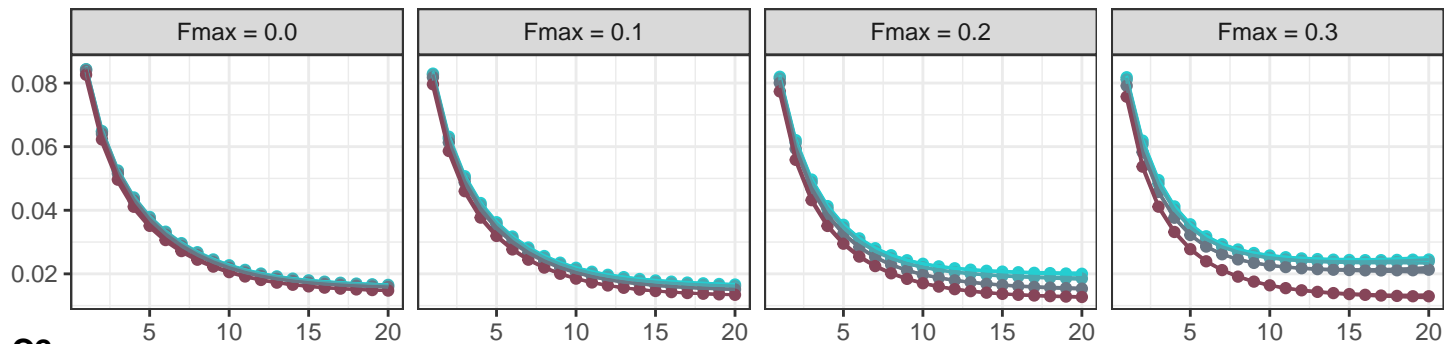

**C2**

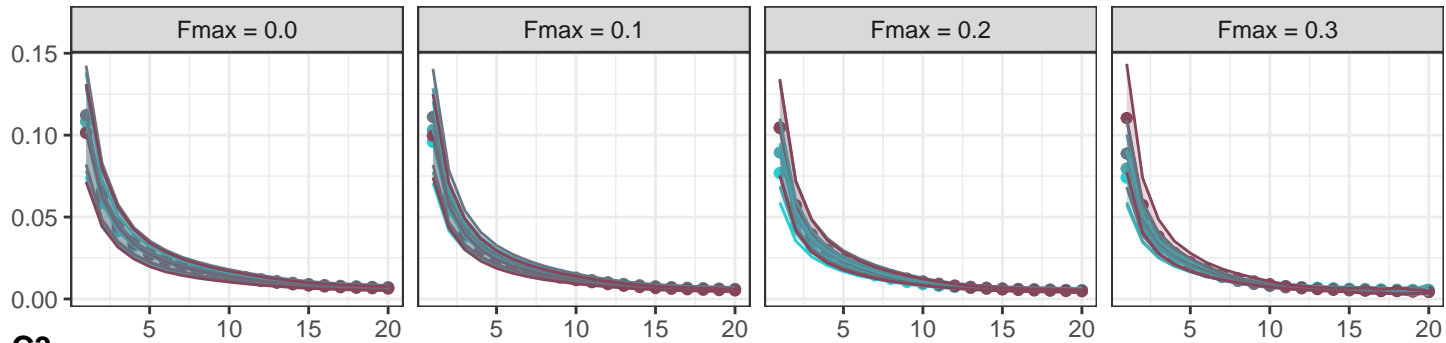

**C3**

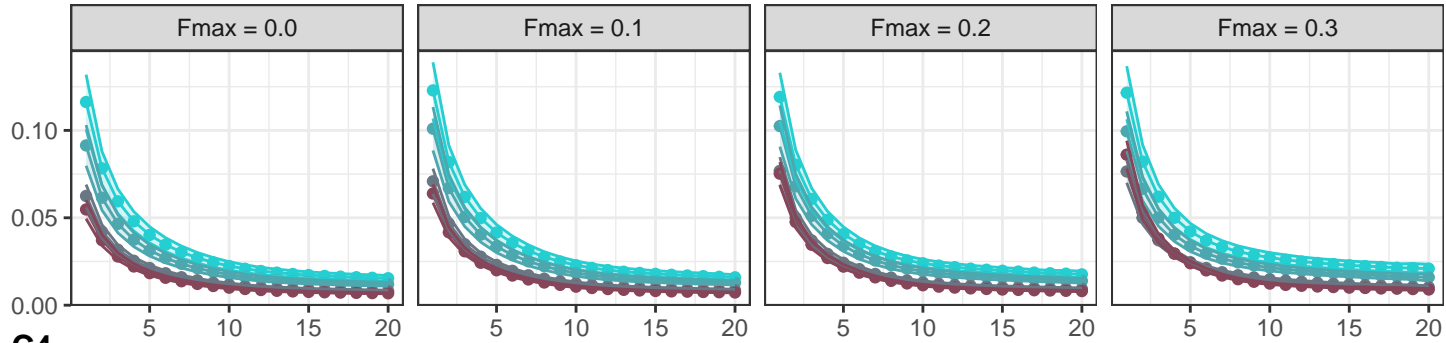

**C4**

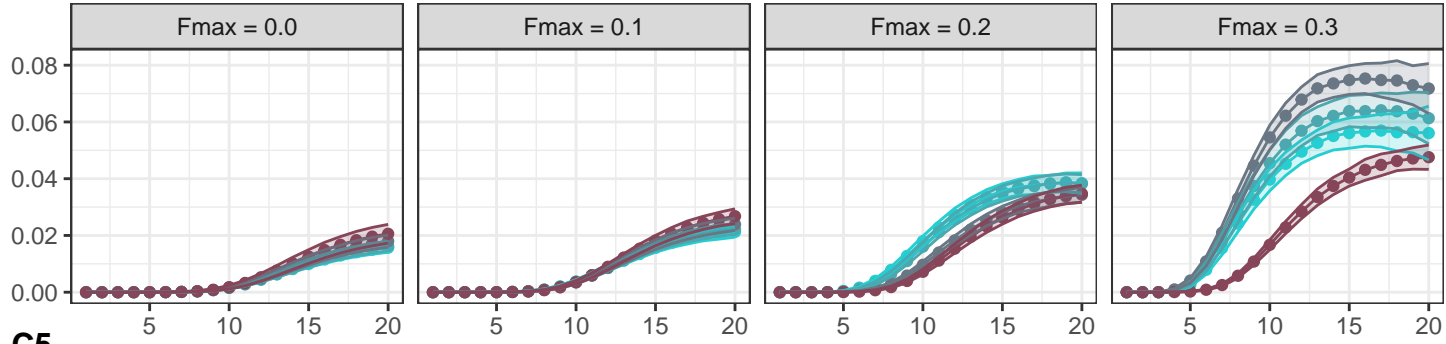

**C5**

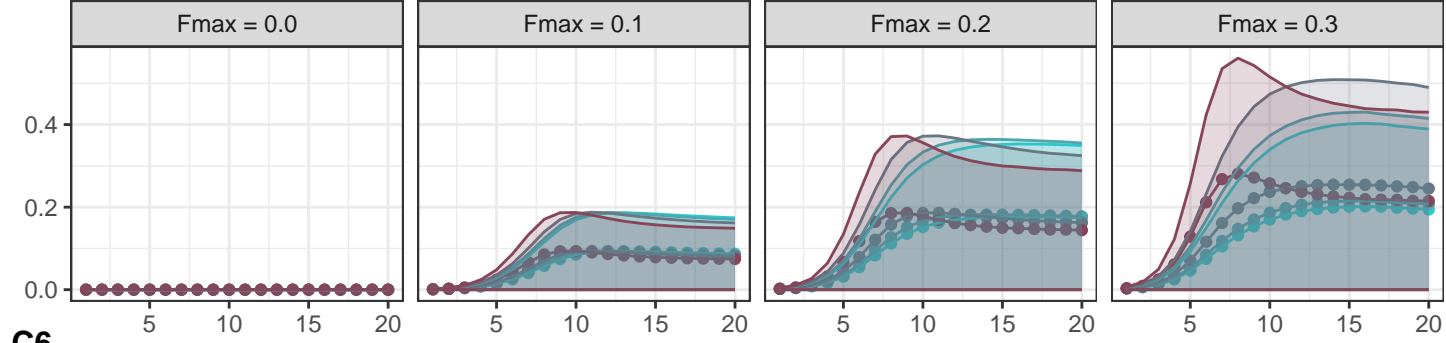

**C6**

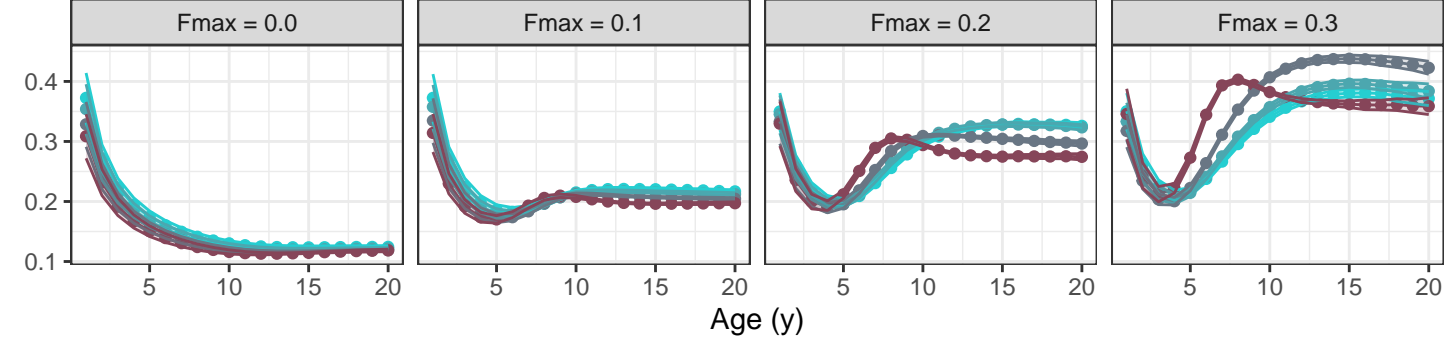

Supplement: Supplementary file 4 — Supplementary Information 4. [file 41598_2025_15394_MOESM4_ESM.pdf]
